# Unfractionated and Low Molecular Weight Heparin Reduce Platelet Induced Epithelial-Mesenchymal Transition in Pancreatic and Prostate Cancer Cells

**DOI:** 10.3390/molecules23102690

**Published:** 2018-10-19

**Authors:** Jan Moritz Ponert, Lukas Maria Gockel, Svenja Henze, Martin Schlesinger

**Affiliations:** Department of Pharmacy, University of Bonn, An der Immenburg 4, 53121 Bonn, Germany; moritz.ponert@uni-bonn.de (J.M.P.); Lukas.Gockel@uni-bonn.de (L.M.G.); svenja.henze@uni-bonn.de (S.H.)

**Keywords:** heparin, platelet, tumor, epithelial-mesenchymal transition, EMT, cancer stem cells, enoxaparin

## Abstract

The interaction with platelets is of crucial importance for tumor cells passing through hematogenous metastasis. Platelets protect cancer cells from immune surveillance and exhibit many other prometastatic effects. Notably, platelets can change the epithelial tumor phenotype, a process termed epithelial-mesenchymal transition (EMT), which confers stem cell-like properties onto tumor cells associated with an increased motility and drug resistance. The aim of the study is to investigate the impact of heparin on the platelet induced EMT program in pancreatic and prostate tumor cells. Platelet activation and interaction with cancer cells were determined by static adhesion assays. Applying ELISAs, the platelet release of EMT inducing mediators was quantified. EMT marker protein expression by tumor cells was explored by western blot and qPCR. Our data show that different tumor cell entities have different platelet binding capacities and also that a weak interaction is sufficient to change tumor cell phenotype. Additionally, unfractionated heparin (UFH) as well as low molecular weight heparin (LMWH) reduced tumor cell platelet interaction. Subsequently, attenuated platelet-derived mediator release resulted in reduced EMT marker protein and transcription factor expression by the cancer cells and decreased cell migration. These data suggest that heparin reduces platelet induced EMT program and prevents the formation of cancer cells with stem cell-like properties. This additional mechanism argues for the use of heparin in oncological applications.

## 1. Introduction

The tumor microenvironment is increasingly regarded as a fundamental regulator of cancer progression and enhanced malignancy. Tumor cells impact their local environment by secretion of diverse bioactive molecules. In turn, host cells, in particular bone marrow-derived cells, crucially affect tumor development, proliferation and finally transmit traits to tumor cell for successful metastatic spread [1,2]. In the hematogenous dissemination, platelets are the first cells tumor cells encounter after invasion [3,4]. Platelets are aggregated by tumor cells and protect tumor cells from shear forces and NK cell lysis [5]. Platelet derived transforming growth factor-β1 (TGF-β1) downregulates activating C-type lectin-like NKG2D receptors on NK cells and platelet derived MHC class I molecules are transferred to tumor cell membranes which confers a pseudonormal tumor phenotype to escape T-cell mediated immune surveillance [6,7]. Platelet derived chemokines CXCL5 and CXCL7 attract granulocytes to the agglomerate of tumor cells and platelets. Recruited granulocytes finally support tumor cell survival and formation of a proper early metastatic niche [8]. Platelet membranes comprise a vast number of adhesion molecules for instance integrins, P-selectin and glycoproteins, which facilitate arrest of the tumor cells at the vascular wall [9,10,11]. Platelet secreted serotonin, ATP or eicosanoids like 12(S)-HETE increase vessel permeability by retraction of endothelial cells and permit tumor cell extravasation to the subendothelial matrix [12,13,14,15,16]. Platelet derived microvesicles induce matrix metalloproteinases expression in tumor cells accompanied by enhanced matrix degradation and tumor cell invasion [17,18]. Finally, platelet-derived growth factors like epidermal growth factor (EGF) or vascular endothelial growth factor (VEGF) stimulate angiogenesis in the nascent metastatic foci and ensure continuous supply with oxygen and nutrition [19,20,21,22]. To pass successfully through all the aforementioned steps, tumor cells must be endowed with an invasive, motile, and mesenchymal phenotype. The program of epithelial-mesenchymal transition (EMT) is characterized by profound biological distinctions in transcription programs affecting cell shape, cytoskeleton proteins and cell-cell adhesions [23,24,25]. Platelets can induce the transition from an epithelial to a mesenchymal phenotype, which finally confers cancer stem cell-like traits to the tumor cells and increases invasiveness [26,27]. Platelet derived TGF-β1 as well as direct contact formation between platelets and tumor cells are indispensable to promote prometastatic gene expression [26]. Cancer cells utilize several pathways to elicit platelet activation and aggregation such as tissue factor expression, ADP or thromboxane A2 release, or integrin ligation among many other mechanisms [28,29]. 

Hence, the procoagulant properties of cancer cells are reflected in the hypercoagulable state of many cancer patients, having a 6–7 fold increased risk of venous thromboembolism [30,31]. Therefore, cancer patients are routinely treated with low-molecular-weight heparin (LMWH) to prevent cardiovascular events. Besides the anticoagulant properties, some clinical trials also revealed prosurvival effects of heparin whereas other trials could not substantiate an impact of heparin on patients’ survival [32,33,34,35]. Notwithstanding, in several in vitro approaches, a multitude of antimetastatic effects for heparin became apparent including blockade of adhesion receptors (like P-and L-selectin or integrin α_IIb_β_III_) or of heparanase, which is expressed by different tumor entities [36,37]. However, whether heparin can also affect the EMT program of tumor cells by platelets has not been shown yet. Thus, in the present study we investigate the impact of unfractionated and low-molecular-weight heparin on platelet induced alteration of tumor cells phenotype. 

Here, we reveal that pancreatic and prostate cancer cells bind to and activate platelets to varying extents. Platelets induce an EMT program in both tumor entities vice versa. Different heparins block the interaction between platelets and tumor cells and mitigate the transition to a mesenchymal phenotype and an associated enhanced invasiveness. Thus, heparin’s many pleitropic effects related to cancer metastasis reveal the importance of heparin in the patients’ treatment regimen.

## 2. Results

### 2.1. Interaction between Different Tumor Cells and Platelets and the Impact of UFH and LMWH

**Figure 1:**
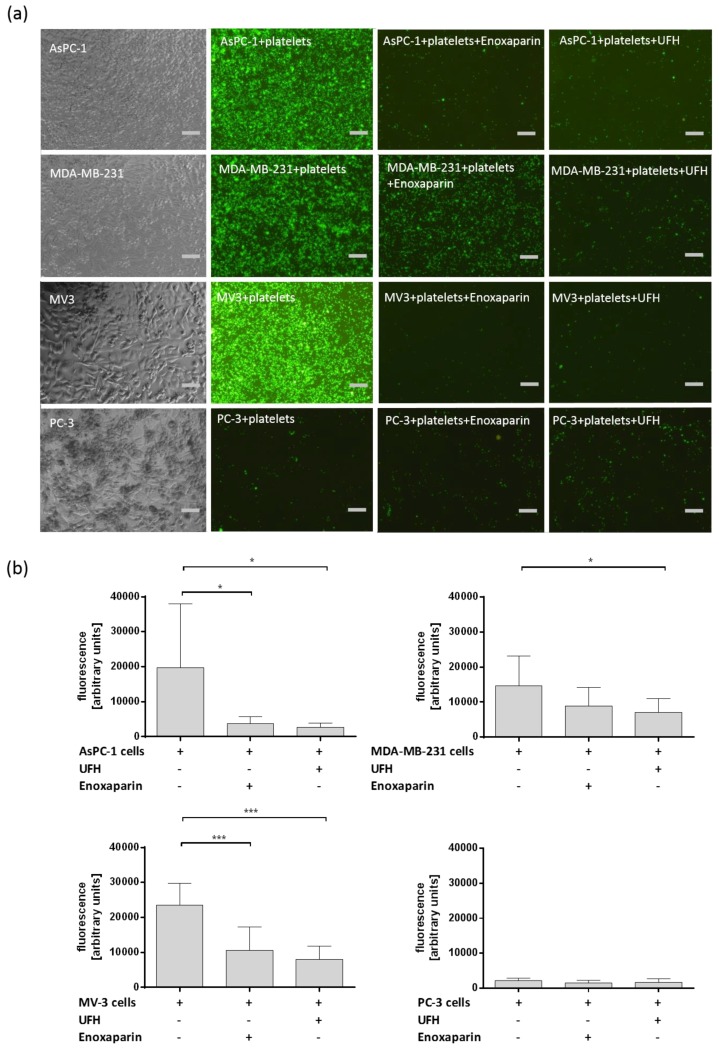
(**a**) Representative pictures of Calcein-AM labeled platelets interacting with confluent layers of AsPC-1, MDA-MB-231, MV3 and PC-3 cells. Prior to interaction platelets were coincubated with 1 IU/mL UFH or 1 IU/mL enoxaparin. Scale bar corresponds to 50 µm. (**b**) Quantification of platelet (Calcein-AM labeled) tumor cell (AsPC-1, MDA-MB-231, MV3 and PC-3) by fluorescent plate reader assay. Data are means of at least *n* = 3 (±SD), asterisks indicate statistical significance: * *p* ≤ 0.05; *** *p* ≤ 0.001.

The binding between tumor cells and platelets is of crucial importance for successful metastatic dissemination of cancer cells by the blood flow. Heparin can interfere in the interaction between tumor cells and platelets. Therefore, direct binding between different tumor cell entities and platelets under static conditions was analyzed with respect to heparin on this interaction. Calcein-AM labeled platelets resuspended in platelet buffer were added to confluent layers of AsPC-1 pancreatic cancer cells, triple-negative human breast cancer cell line MDA-MB-231, human melanoma cell line MV3, or prostate cancer cell line PC-3, respectively. AsPC-1, MDA-MB-231, and MV3 cells exhibited a strong interaction with platelets whereas PC-3 cells revealed merely a weak interplay with platelets (Figure 1a,b). Platelets previously incubated with UFH or LMWH enoxaparin featured a reduced adhesion to confluent cell layers compared to untreated platelets whereas binding of platelets to PC-3 prostate cancer cells was hardly affected by UFH or enoxaparin pretreatment (Figure 1). Different tumor cells are obviously able to activate platelets by direct contact formation which expedites platelet tumor cell adhesion. AsPC-1 cells exhibit pronounced platelet activating capacities and PC-3 cells showed a rather weak tendency for platelet activation and subsequent adhesion. 

### 2.2. Impact of AsPC-1 and PC-3 Cell Induced Platelet Activation on Hepatocyte Growth Factor (HGF) and Platelet-Derived Growth Factor (PDGF) Granule Secretion

**Figure 2:**
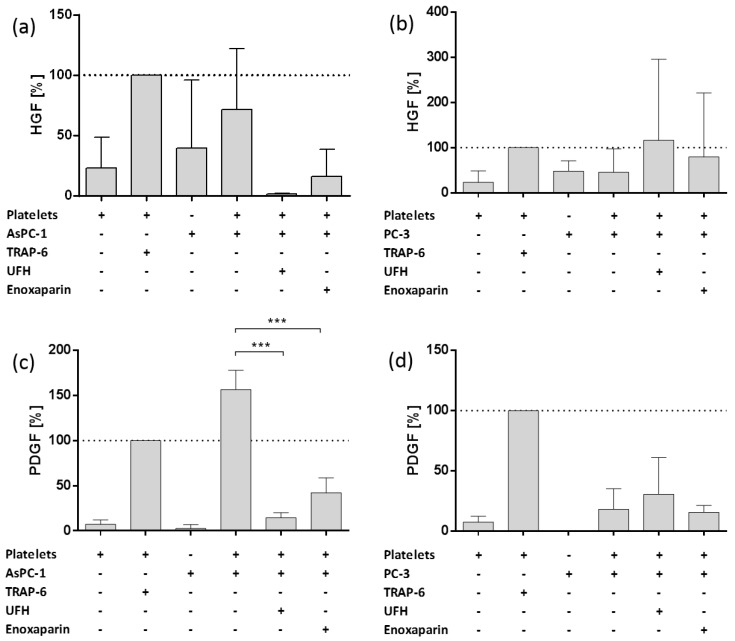
Impact of heparin on platelet derived HGF and PDGF release. (**a**) Impact of UFH or Enoxaparin on AsPC-1 cell induced HGF release from platelets. (**b**) Impact of UFH or enoxaparin on PC-3 cell induced HGF release from platelets. (**c**) Impact of UFH or enoxaparin on AsPC-1 cell induced PDGF release from platelets. (**d**) Impact of UFH or enoxaparin on PC-3 cell induced PDGF release from platelets. Data are means of at least *n* = 3 (±SD), asterisks indicate statistical significance: *** *p* ≤ 0.001.

To elucidate the effect of direct platelet tumor cell interaction on the formation of a potential metastatic niche, we analyzed platelets’ α-granules release due to cancer cell interaction. For this reason, we quantified Hepatocyte growth factor (HGF) and Platelet-derived growth factor (PDGF) secretion from platelets with ELISAs. We selected AsPC-1 cells with strong and PC-3 cell line with rather weak platelet interaction capacities. Platelets activated with thrombin receptor activator peptide 6 (TRAP-6), as ligand for platelets’ PAR-1 receptor, exhibited a pronounced HGF release compared to resting platelets or AsPC-1 or PC-3 cells alone, respectively (Figure 2a,b). Platelets coincubated with AsPC-1 cells revealed a similar HGF release like mediated by TRAP-6 (Figure 2a). This effect was susceptible to UFH and enoxaparin incubation, since UFH completely inhibited HGF release and enoxaparin reduced HGF concentration to 20% compared to secretion induced by TRAP-6. In contrast, PC-3 cells induced only 50% of HGF secretion in comparison to TRAP-6 and the secretion was not prone to a UFH or enoxaparin inhibition. Both heparins rather increased HGF release from platelets′ α-granules (Figure 2b). Both cell lines exhibit similar release characteristics for PDGF release (Figure 2c,d). AsPC-1 cells induced a stronger PDGF release from platelets than TRAP-6 and UFH as well as enoxaparin reduced PDGF release to 15% and 40%, respectively (Figure 2c). PC-3 cells were again unable to induce intense PDGF secretion and also UFH and enoxaparin had no inhibitory impact on PC-3 mediated PDGF release (Figure 2d).

### 2.3. Impact of AsPC-1 and PC-3 Cell Induced Platelet Activation on Epidermal Growth Factor and Transforming Growth Factor Beta 1 Granule Release

**Figure 3:**
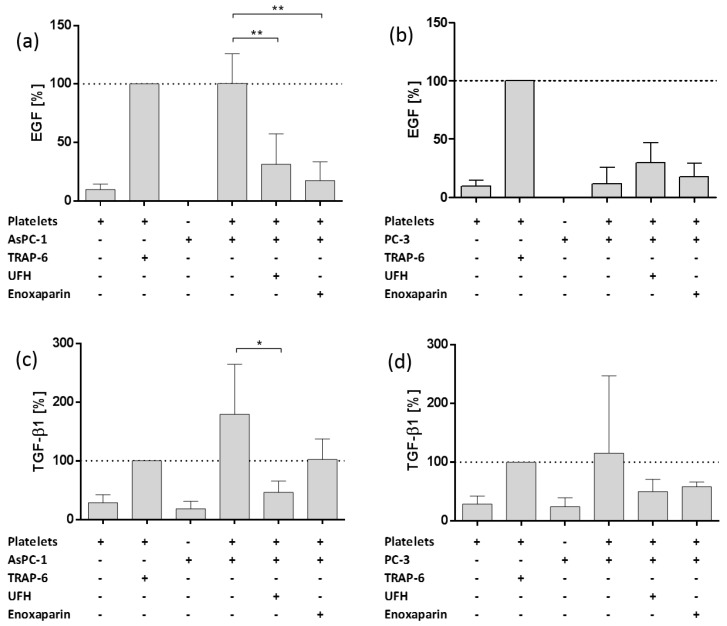
Impact of heparin on platelet derived EGF and TGF-β1 release. (**a**) Impact of UFH or enoxaparin on AsPC-1 cell induced EGF release from platelets. (**b**) Impact of UFH or enoxaparin on PC-3 cell induced EGF release from platelets. (**c**) Impact of UFH or enoxaparin on AsPC-1 cell induced TGF-β1 release from platelets. (**d**) Impact of UFH or enoxaparin on PC-3 cell induced TGF-β1 release from platelets Data are means of at least *n* = 3 (±SD), asterisks indicate statistical significance: * *p* ≤ 0.05; ** *p* ≤ 0.01.

After quantification of growth factor release, next, we investigated the impact of AsPC-1 and PC-3 cells on EMT inductor secretion from platelets’ α-granules. Epidermal growth factor (EGF) and Transforming growth factor beta 1 (TGF-β1) act as potent drivers of cancer progression through the induction of epithelial-mesenchymal transition (EMT), in which epithelial cells acquire a mesenchymal phenotype and gain cancer stem-cell-like properties [38]. AsPC-1 cells induced EGF release similar to TRAP-6 addition and UFH and enoxaparin potently attenuated EGF secretion due to AsPC-1 administration (Figure 3a). PC-3 cells in turn merely induced a slight EGF release from platelets compared to TRAP-6. UFH as well as enoxaparin had no impact on EGF secretion, actually EGF concentrations were negligibly increased by both heparins (Figure 3b). For TGF-β1, AsPC-1 cells initiated a severe release from platelets’ granules, which was even higher than TGF-β1 release induced by TRAP-6 (Figure 3c). UFH as well as enoxaparin profoundly reduced TGF-β1 secretion. Surprisingly, PC-3 cells exhibited remarkable endogenous TGF-β1 release but were unable to induce TGF-β1 secretion from platelets (Figure 3d). UFH and enoxaparin, respectively, again showed an activating effect on TGF-β1 release when coincubated with PC-3 cells and platelets (Figure 3d).

### 2.4. Interaction of Platelets with Tumor Cells Induces a Mesenchymal Phenotype

**Figure 4:**
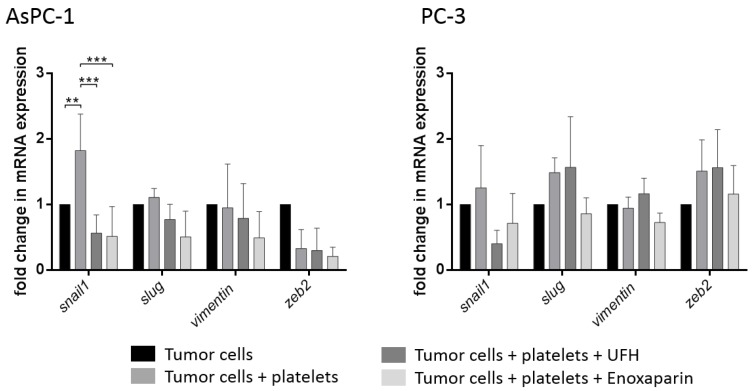
Relative fold change in mRNA expression in AsPC-1 or PC-3 cells treated with buffer, platelets, platelets and UFH, or platelets and enoxaparin for 72 h, respectively. Values are normalized to Gapdh, β-actin and RPL13 expression. Statistical significance was evaluated by two-way ANOVA test. Data are means of at least *n* = 3 (±SD), asterisks indicate statistical significance: ** *p* ≤ 0.01; *** *p* ≤ 0.001.

Since AsPC-1 cells are capable of inducing a potent release of growth factors and molecules with EMT inducing properties, we next sought to determine the impact on tumor cell mesenchymal markers and transcription factors involved in EMT. Real-Time PCR analysis of the transcription factors snail, slug, vimentin, and zeb2 revealed a significant upregulation of mRNA levels of Snail and a slight upregulation of vimentin in AsPC-1 cells due to platelet contact for 72 h (Figure 4). Levels of snail and vimentin were reduced by means of UFH or enoxaparin coincubation. Surprisingly, treatment of PC-3 cells with platelets for 72 h induced an upregulation of snail, slug, and zeb2 mRNA levels. Although only a weak interaction and granule release from platelets was detectable for PC-3 cells, the crosstalk is obviously sufficient to change gene expression of the PC-3 cells at least partially to a more mesenchymal phenotype. Coincubation of PC-3 cells with UFH reduced snail mRNA levels and enoxaparin even downregulated snail, slug, vimentin and zeb2 mRNA expression (Figure 4).

**Figure 5:**
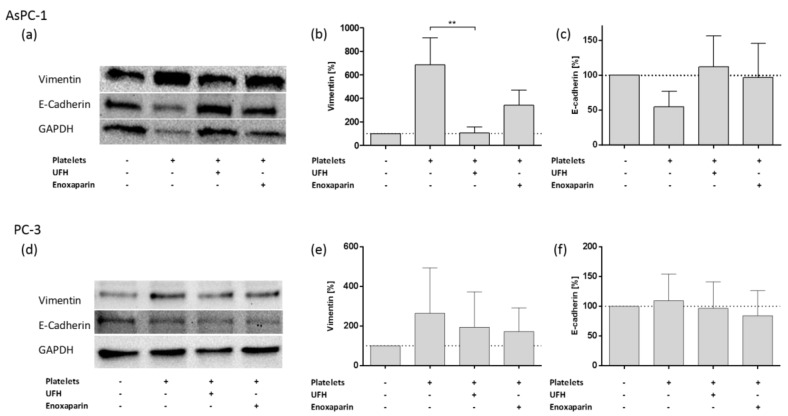
Western blotting of the expression profile of E-cadherin and vimentin in AsPC-1 and PC-3 cells. (**a**) Expression of E-cadherin and vimentin in AsPC-1 cells after treatment with platelets or platelets and UFH or platelets and enoxaparin for 72 h, respectively. (**b**) Quantification of vimentin expression by pixel density measurements in AsPC-1 cells, data of three independent experiments. Asterisks indicate statistical significance: ** *p* ≤ 0.01. (**c**) Quantification of E-cadherin expression by pixel density measurements in AsPC-1 cells, data of three independent experiments. (**d**) Expression of E-cadherin and vimentin in PC-3 cells after treatment with platelets or platelets and UFH or platelets and enoxaparin for 72 h. (**e**) Quantification of vimentin expression by pixel density measurements in PC-3 cells, data of three independent experiments. (**f**) Quantification of E-cadherin expression by pixel density measurements in AsPC-1 cells, data of three independent experiments.

To confirm the results obtained by Real-Time PCR and to focus on an epithelial marker, we detected E-cadherin and vimentin protein levels by immunoblotting of lysates of AsPC-1 and PC-3 cells, respectively (Figure 5). AsPC-1 cells revealed an increased expression of vimentin and downregulation of the epithelial protein E-cadherin due to platelet coincubation (Figure 5a–c). UFH and enoxaparin treatment inverted the platelet induced mesenchymal phenotype in AsPC-1 cells and alleviated vimentin protein levels and enhanced E-cadherin expression. For PC-3 cells, which exhibit weak platelet interactions, only an increase in vimentin protein expression was observable and heparin treatment reduced vimentin levels (Figure 5d,e). Pixel density analysis of western blot bands uncovered a slight E-cadherin upregulation in PC-3 cells due to platelet coincubation and no impact of UFH as well as enoxaparin addition on E-cadherin levels, respectively (Figure 5f). Although, EMT related gene expression clearly pinpoint to a mesenchymal phenotype in platelet treated PC-3 cells, the classical protein EMT marker E-cadherin does not indicate a change in phenotype for PC-3 cancer cells. 

### 2.5. Platelet Treatement Induces an Invasive and Promigratory Phenotype in Tumor Cells

**Figure 6:**
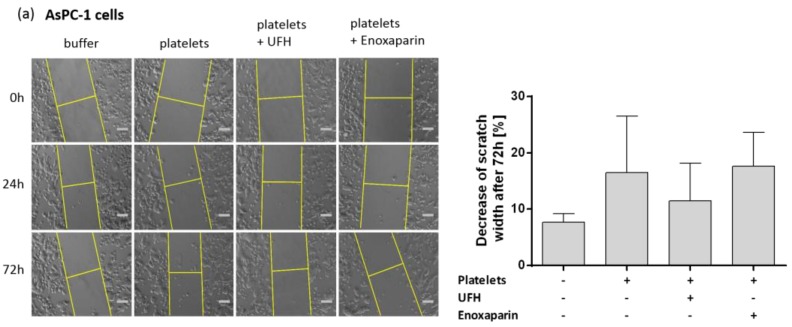

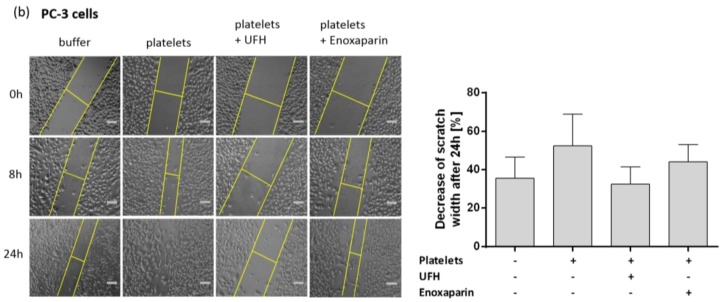
Representative images from wound healing assays of AsPC-1 cell and PC-3 cell cultures treated with buffer or platelets or platelets and heparins. Scale bar corresponds to 50 µm. Quantification of scratch width decrease in percent is based on data of three independent experiments. (**a**) Wound healing assay after 72 h coincubation of AsPC-1 cells with buffer, platelets, platelets and UFH, or platelets and enoxaparin and 16 h serum-starvation. Images show the invasion of AsPC-1 cells into the cell-free region at the beginning (0 h), after 24 h and after 72 h. Histogram shows the decrease of the scratch size after 72 h (right part of the figure). (**b**) Wound healing assay after 72 h coincubation of PC-3 cells with buffer, platelets, platelets and UFH or platelets and enoxaparin and 16 h serum-starvation. Images show the invasion of PC-3 cells into the cell-free region at the beginning (0 h), after 8 h and after 24 h. Histogram shows the decrease of the scratch size after 24 h (right part of the figure).

To directly define whether tumor cells with mesenchymal traits promote an invasive and promigratory behavior, we performed scratch wound healing assays. Platelet treated AsPC-1 cells for 72 h exhibited an increased migration after 48 h compared to untreated cells (Figure 6a). Coincubation of UFH with tumor cells and platelets reduced cell migration and the scratch size decrease was attenuated after 72 h. Thus, UFH is obviously capable of inhibiting a platelet induced EMT in pancreatic cancer cells. Enoxaparin has no impact on tumor cell migration. 

Surprisingly, PC-3 cells previously treated with platelets for 72 h, displayed an invasive phenotype compared to untreated PC-3 cells and the scissure in the cell layer was almost completely closed after 24 h. UFH coincubation also reduced invasive behavior of the PC-3 cells and closing of the scissure was delayed to a comparable level of untreated PC-3 cells (Figure 6b). Enoxaparin expresses solely a slight inhibitory capacity on tumor cell migration.

## 3. Discussion 

In this work, we provide evidence that UFH and LMWH reduce the interaction of platelets with pancreatic and prostate cancer cells and thereby constrain platelet mediated tumor cell transition from an epithelial to a mesenchymal phenotype. UFH as well as LMWH severely impact release characteristics of EMT inductors from α-granules in pancreatic tumor cells, which is apparently responsible for a change in tumor cell phenotype. In contrast, prostate cancer cells exhibiting only slight interactions with platelets and are hardly able to activate platelets. Nonetheless, weak contact is adequate to modify prostate cancer cell phenotype by induction of an EMT program with increased invasiveness. This is an impressive finding that the malignancy of tumors is obviously dictated by the communication with platelets whereas the intensity of the interaction is apparently not decisive for successful transit through an EMT program. It is a well-known fact that heparin is able to reduce the interaction of tumor cells with platelets and leukocytes [39,40]. Several adhesion receptors have been revealed as potential targets for heparin to reduce tumor cell induced agglomerate formation in the blood. P-selectin as well as integrin α_IIb_β_III_ mediate tumor cell platelet interactions and L-selectin finally recruits leukocytes to the growing embolus [41,42,43]. It is tempting to speculate that these receptors may be potentially responsible for the reduced interaction of tumor cells with platelets due to heparin application and account for the attenuated release characteristics of EMT relevant mediators. Heparin obviously affects both the direct interaction of cancer cells with platelets and the secretion of bioactive molecules from the α-granules of the platelets. These results are in line with Battinelli et al. who established that a heparin induced shift from a pro- to an antiangiogenic secretome was induced in breast cancer cells [44]. 

Our data demonstrate that this heparin feature is also valid for proteins with EMT inducing capacities. Nonetheless, it is not completely clear whether a direct interaction between cancer cells and platelets or the release of EMT inducing molecules causes the shift from an epithelial to a mesenchymal phenotype. It is also conceivable that a combination of both mechanisms acts synergistically in changing tumor cell traits. The trans-differentiation program of EMT is closely associated with the generation of tumor cells with stem-cell like properties [23]. Cancer cells that have gone at least partially through an EMT program lose their differentiated properties of cell to cell contacts and lack of motility instead attaining properties of mesenchymal cells such as increased invasiveness and resistance to cytotoxic drugs [45]. In carcinomas the EMT program is induced by convergence of various signals from the tumor stroma like collagen or host cells such as fibroblasts [46], neutrophils [47], monocytes and macrophages [48,49], mesenchymal stem cells [50] and also platelets [26] in a paracrine or juxtacrine fashion [25]. Cancer cells that encounter a combination of different signaling molecules like PDGF [51], HGF [52], TGF-β1 [49,53], or EGF [54] among many others secreted by the complex tumor microenvironment orchestrate the gene expression of cancer cells for instance by upregulation of transcription factors like *snail*, *twist*, or *zeb1* [55]. This ultimately culminates in changes in cancer cell plasticity concerning cell morphology, polarity and motility [25]. For pancreatic and prostate cancer cells different microenvironmental cues like TGF-β1, irradiation or interaction with platelets have been identified which instigate variants of EMT programs [56,57,58]. Our current data show that AsPC-1 cells have the ability to activate platelets, which vice versa induce a mesenchymal phenotype. This interaction is susceptible to heparin application. PC-3 prostate cancer cells exhibit diminished platelet binding compared to AsPC-1 cells but this is finally sufficient to induce a mesenchymal and invasive phenotype. UFH as well as LMWH application reduce platelet mediated EMT in PC-3 cells. 

These findings finally add a novel mechanism of heparin in the hematogenous metastatic spread of tumor cells with regard to cancer stem cell formation. Stem cell properties can apparently support tumor cell survival, extravasation, and finally seeding in a distant organ and make the inefficient process of metastasis more efficient. Nonetheless the positive clinical effects of heparin for cancer patients beyond anticoagulation are far from being clear since several prospective trials could determine a heparin related pro-survival benefit whereas other trials could not ascertain any beneficial outcomes [32,33,59,60]. Obviously, patients at an early stage of disease seem to profit from heparin administration, in contrast, cancer patients at a late stage with severe progression do not benefit from heparin treatment. Thus, additional trials are urgently needed to elucidate the clinical status of heparin especially in the light of the multiple inhibitory effects on the molecular level of heparin on cancer progression and metastasis. 

## 4. Materials and Methods 

### 4.1. Cell Lines 

Human AsPC-1 pancreas carcinoma cells and human MV3 melanoma were maintained in RPMI 1640 medium (PAN Biotech, Aidenbach, Germany) containing 10% (*v*/*v*) fetal calf serum (FCS, Sigma Aldrich, Steinheim, Germany), 100 U/mL penicillin and 100 μg/mL streptomycin (PAN Biotech). MDA-MB-231 breast cancer cells were cultivated in DMEM (high glucose) medium (PAN Biotech) and supplemented with 10% (*v*/*v*) FCS, 1% (*v*/*v*) l-glutamine (PAN Biotech), 100 U/mL penicillin and 100 μg/mL streptomycin. PC-3 prostate cancer cells were cultivated in RPMI 1640 medium containing 10% (*v*/*v*) FCS, 100 U/mL penicillin and 100 μg/mL streptomycin, 1% (*v*/*v*) l-glutamine and 1% (*v*/*v*) sodium pyruvate (Thermo Fisher Scientific, Waltham, MA, USA). All cell lines were incubated at 37 °C in a humidified atmosphere containing 5% (*v*/*v*) CO_2_. For subcultivation, all cell lines were incubated with trypsin/EDTA (5 g/L trypsin; 0.2 g/L EDTA × tetra sodium, Sigma Aldrich) for 5 min at 37 °C. Mycoplasm check was conducted every four weeks.

### 4.2. Platelet Isolation and Activation

Platelets were obtained from one day expired human platelet-rich plasma concentrates (anticoagulated with citrate) by centrifugation at 670× *g* for 10 min. Isolated platelet pellets were resuspended in platelet buffer (10 mM *N*-2-hydroxyethylpiperazine-*N*9-2-ethanesulfonic acid, 140 mM NaCl, 3 mM KCl, 0.5 mM MgCl_2_, 5 mM NaHCO_3_, 10 mM glucose) and the concentration was adjusted to 4 × 10^8^ platelets/mL. CaCl_2_ was added to a concentration of 1 mM. Prior to activation, in some cases preincubation of platelets was carried out with UFH (UFH, Ratiopharm GmbH, Ulm, Germany) and Enoxaparin (Sanofi, Paris, France) for 30 min. The anticoagulant exposure was performed in platelet buffer at a final concentration of 1 IU/mL which corresponds to therapeutic concentrations in anticoagulated patients. Platelets were stimulated with 42.5 µM Thrombin Receptor Activating Peptide-6 (TRAP-6) (Tocris Bioscience, Bristol, UK) or 1 × 10^4^ tumor cells/mL for 12 min at 37 °C. Activation by tumor cells was carried out with PC-3 and AsPC-1 cells in presence or absence of anticoagulants. Platelet supernatants/releasates were obtained by sample centrifugation for 20 min at 1000× *g*.

### 4.3. EMT Induction

For EMT induction, tumor cells with 90% confluence were incubated with 5 mL platelet rich buffer (4 × 10^8^ platelets/mL platelet buffer) and 5 mL medium for 72 h to obtain a final and physiological platelet concentration of 2 × 10^8^ platelets/mL. In some cases, platelets were incubated for 30 min with UFH and enoxaparin (1 IU/mL). Tumor cell viability was checked daily by microscopy and trypan blue staining. After 72 h, cells were washed five times with PBS, carefully detached with a cell scraper, pelletized by centrifugation and stored by −80°C for western blot and qRT-PCR.

### 4.4. Adhesion Assay

For quantification of adhesive interactions between tumor cells and platelets, tumor cells were cultivated in 96-well plates to confluence. Platelets isolated by centrifugation and resuspended in platelet buffer were incubated with Calcein-AM (2 µM) (Sigma Aldrich) for 15 min at 37 °C. Calcein-AM labeled platelets were washed twice with warm PBS (37 °C) resuspended in platelet buffer and coincubated for 30 min with UFH or enoxaparin (1 IU/mL final concentration). Subsequently, 4 × 10^7^ Calcein-AM-labeled platelets/100 μL platelet buffer were administered. After 20 min of incubation, unbound platelets were removed. Then tumor cell bound platelets were lysed with 100 μL Triton X-100 (10% in PBS, Sigma Aldrich). Fluorescence was quantified at an emission wavelength of 520 nm applying a fluorescence plate reader (BMG POLARstar, BMG Labtech, Ortenberg, Germany).

### 4.5. ELISA 

The PDGF, EGF, HGF, and TGF-β1 concentrations of the platelet releasates were determined with human enzyme-linked immunosorbent assays according to the manufacturer’s instructions (PDGF, EGF; PeproTech, Rocky Hill, CT, USA, HGF; Ray Biotech, Norcross, GA, USA, TGF-β1; Thermo Fisher Scientific).

### 4.6. qRT-PCR

The RNA was extracted using the Direct-Zol™ RNA MiniPrep kit (Zymo Research, Irvine, CA, USA) according to the manufacturer’s instructions. The total RNA amount was quantified with a Colibri Microvolume Spectrometer (Titertek-Berthold, Wildbad, Germany). Sample RNA concentration was adjusted to 2 µg/20µL and contained DNA was digested with the DNase I, RNase-free (1 U/µL) kit purchased from Thermo Fisher Scientific and used according to the manufacturer’s instructions. Afterwards purified samples were separated into two 1 µg/10 µL samples and used for reverse transcription, which was carried out using the iScript™ Advanced cDNA Synthesis kit (BioRad, Hercules, CA, USA) according to the manufacturer’s instructions. Each qRT-PCR reaction was performed with 5 ng sample DNA and as master mix the Sso™ Advanced Universal SYBR^®^ Green Supermix (BioRad) was applied. The following primers were used for qRT-PCR reactions *snail*, *slug* (BioRad), *vimentin* and *zeb2* (Biomol, Hamburg, Germany). All reactions were performed in duplicate with thermal cycling at 95 °C for 3 min, 50 cycles of 95 °C for 20 s and 60 °C for 20 s and 68 °C for 40 s by a DNA Engine Opticon™ System (BioRad, Hercules, CA, USA, formerly MJ Research). The mean CT values of each sample were calculated by the Opticon™ monitor analysis software (version 1.08, Biorad, formerly MJ Research). AsPC-1 and PC-3 cell sample normalizations were performed using the housekeeping genes glyceraldehyde 3-phosphate dehydrogenase (Gapdh), β-actin (BioRad) and ribosomal protein L13 (RPL13) (Biomol). Relative quantification of mRNA expression was calculated by the 2^−ΔΔ*C*T^ method.

### 4.7. Western Blot 

First, cell pellets obtained by EMT induction experiments were lysed using extraction buffer (Life Technologies, Carlsbad, CA, USA), supplemented with protease inhibitor cocktail (1 g/mL aprotinin, 1 g/mL leupeptin) (Life Technologies) and phenylmethanesulfonylfluoride (0.1 mM PMSF) (Life Technologies), according to manufacturer’s instructions. Then supernatants were collected by centrifugation (35,000× *g*, 15 min, 4 °C) and protein quantification was carried out using a Pierce™ BCA Protein Assay Kit (Thermo Fisher Scientific). SDS/PAGE was performed with 25 µg protein for each sample using precast gels in a polymerization degree of 10% (Mini-PROTEAN^®^ TGX™ Stain-Free™; BioRad). Afterwards proteins were transferred to a Roti^®^-PVDF membranes (Carl Roth GmbH, Karlsruhe, Germany). Membranes were blocked for 60 min with a solution of milk powder in Tris-buffered saline-Tween 20 (TBS-T) (5% milk powder, 0.2% Tween 20) and washed thrice with TBS-T (2% Tween 20). Then membranes were incubated with the primary antibodies mouse anti-E-cadherin and mouse anti-vimentin (Santa Cruz Biotechnology, Dallas, TX, USA) both diluted 1:200 and the primary antibody mouse anti-Gapdh (GeneTex, Irvine, CA, USA) for 60 min at room temperature followed by an incubation overnight at 4 °C. Afterwards, primary antibodies were removed and membranes were washed again three times with TBS-T. Then membranes were incubated with the secondary antibody anti-mouse m-IgGκ HRP-conjugated (Santa Cruz) diluted 1:2000 for 90 min. At last, membranes were detected via chemiluminescence using Clarity Western ECL substrate chemiluminescence kit (BioRad) and membranes were photographed and quantified using ChemiDoc XRS+ imaging acquiring system and Image Lab software (version 5.2.1, both from BioRad). As housekeeping protein for normalization glyceraldehyde 3-phosphate dehydrogenase (Gapdh) was used. 

### 4.8. Wound Healing/Scratch Assay

Scratch assay was performed as described before. Briefly, 1 × 10^6^ AsPC-1 and PC-3 cells either untreated or treated with platelets and heparin for 72 h were grown on uncoated 6 well plates to confluency (STARLAB GmbH, Hamburg, Germany). After 16 h serum-starvation a scratch was conducted with a 10 μL pipette tip (STARLAB GmbH, Hamburg, Germany) and images were taken after the indicated hours. For quantification of the scratch width decrease, distance between the cells was measured at three different spots after the indicated incubation time and mean values were used for calculation. 

### 4.9. Statistical Analysis

Data represent means ± standard deviations of at least three independent experiments if not indicated otherwise. Student’s *t*-test or two-way ANOVA test (for qRT-PCR data) were used for statistical analysis. *p* < 0.05 was regarded as statistically significant and marked with a star. Two stars indicated a *p*-value below 0.01 and three stars were used for *p*-values below 0.001.

## 5. Conclusions

Cancer stem cells are currently regarded as the key players in the process of metastasis, chemoresistance, and clinical relapse due to their capacities to switch between a mesenchymal (cancer stem cell state) and epithelial state, which is on the one hand associated with increased resistance to existing treatments and on the other with rapid proliferation. Drugs with the ability to specifically target cancer stem cells are urgently needed and may become the next milestone in the battle against cancer. Our current data allow the conclusion that UFH as well as LMWH, applied in therapeutic concentrations, obliterate or at least partially reduce the EMT program in pancreatic as well as prostate cancer cells. For AsPC-1 cells, attenuated binding and mitigated bioactive mediator release seem to be responsible for diminished EMT marker expression, reduced invasiveness and ultimately prevented stem cell formation. In contrast, PC-3 cells are obviously poor platelet activators but the weak interplay with platelets appears to be sufficient for a shift to a mesenchymal and invasive phenotype. Different cancer entities require variable signal intensities for transition through an EMT program associated with stem cell-like properties. Here, we add a novel mechanism to the list of heparin′s anti-metastatic and anti-tumorigenic activities concerning cancer stem cell formation.
